# Association between Gut Microbiota Dysbiosis and the CHA2DS2-VASc Score in Atrial Fibrillation Patients

**DOI:** 10.1155/2022/7942605

**Published:** 2022-05-06

**Authors:** Chen Fang, Kun Zuo, Wanjing Zhang, Jiuchang Zhong, Jing Li, Li Xu, Xinchun Yang

**Affiliations:** Heart Center & Beijing Key Laboratory of Hypertension, Beijing Chaoyang Hospital, Capital Medical University, Beijing 100020, China

## Abstract

**Background:**

In our previous studies, we found a disordered taxonomic composition and function of gut microbiota (GM) in atrial fibrillation (AF) patients. However, direct evidence about the association between dysbiotic microbiota and thromboembolic risk in AF is lacking.

**Aims:**

In this study, we analyzed the interaction of GM and related functional patterns in AF with different CHA2DS2-VASc scores to assess its potential as a biomarker for predicting stroke risk. *Patients and Methods*. The CHA2DS2-VASc score was used for thromboembolic risk stratification in AF according to American Heart Association (AHA) guidelines. We investigated the taxonomic and functional annotation of GM based on metagenomic data from 50 AF patients (32 with high thromboembolic risk (CHA2DS2-VASc score ≥2 (males) or CHA2DS2-VASc score ≥3 (females)) and 18 individuals with low thromboembolic risk (CHA2DS2-VASc score <2 (males) or CHA2DS2-VASc score <3 (females))).

**Results:**

The gut microbial diversity, composition, and function in AF were different in high and low CHA2DS2-VASc score groups. In high thromboembolic risk group, the abundance of *Prevotella, Lachnospiraceae,* and *Eubacterium rectale,* related to the production of short-chain fatty acids and anti-inflammatory were reduced (all *P* < 0.05). Furthermore, annotated by Kyoto Encyclopedia of Genes and Genomes (KEGG), a database of genes and genomes, the KEGG orthology-based scoring approach exhibited a significant association with thromboembolic risk in AF patients.

**Conclusions:**

Imbalance of GM and microbial dysfunction are involved in aggravated thromboembolic risk of AF.

## 1. Introduction

Atrial fibrillation (AF), the most frequent cardiac arrhythmia in adults, increases the risk of cerebral and systemic thromboembolic events and is associated with increased morbidity and mortality [1]. Up till now, CHA2DS2-VASc (congestive heart failure (CHF), hypertension (HTN), age ≥75 years, diabetes mellitus (DM), stroke, vascular disease, age 65 to 74 years, female sex) has been the most widely used thromboembolic risk prediction score in patients with AF. According to large observational studies, patients with greater scores, CHA2DS2-VASc score ≥2 (males) or CHA2DS2-VASc score ≥3 (females), have high risks of embolism and are recommended to receive prevention treatment [1]. However, oral anticoagulants are also associated with bleeding events.

Increasing evidence shows an association between the gut microbiota (GM) and cardiovascular diseases, including atherosclerosis, dyslipidemia, HTN, and heart failure [2–7]. The GM can affect host immunomodulatory function and cardiovascular health by producing bioactive metabolites, such as amino acids, peptides, lipopolysaccharides (LPS), trimethylamine-N-oxide (TMAO), and bile acids [5–8]. Our previous studies found that dysbiosis of GM and metabolic patterns is associated with the development and types of AF [2, 9, 10]. However, whether the composition of GM can impact stroke risks remains unexplored. In this study, we analyzed the interaction of GM and related functional patterns in AF patients with different CHA2DS2-VASc scores to assess the relationship between disordered GM and thromboembolic risk in AF patients.

## 2. Patients and Methods

### 2.1. Study Cohort

Fifty nonvalvular AF patients were included in our previous study [2] According to history and personal information, patients were divided into two groups: low (*n* = 18) CHA2DS2-VASc score group and high (*n* = 32) CHA2DS2-VASc score group. The CHA2DS2-VASc score was defined as follows: CHF, HTN, age 65 to 74 years, DM, history of vascular disease and female (1 point), age ≥75 years, and history of stroke (2 points). All patients received a standardized evaluation, including face-to-face surveys, physical examination, 12-lead electrocardiogram, blood chemistry, and echocardiography. The study had approval from the Ethics Committee of Beijing Chaoyang Hospital. The research protocol conformed to principles of the Declaration of Helsinki. All subjects were enrolled at informed consents.

### 2.2. Assessment on GM Composition

The whole metagenome sequencing data of 50 feces samples used in the present study were available from our previous study [2]. Metagenomic analyses were performed as we previously described [2, 3, 9, 10]. Detailed processes are shown in the supplementary methods (Additional file 1).

Two parameters of GM composition, including Pielou evenness and Shannon diversity, were assessed. Furthermore, all samples were clustered via partitioning around medoid (PAM) clustering methods and principal coordinate (PCoA) analysis based on the Jensen–Shannon distance [2]. The linear discriminant analysis (LDA) with effect size measurements (LEfSe) were used to identify differentially abundant bacterial taxa among groups.

### 2.3. Construction and Validation of a Predictive Model for Risks of Embolism in AF Patients

The most useful predictive indexes between AF patients with low or high CHA2DS2-VASc scores were selected by the least absolute shrinkage and selection operator (LASSO) analysis as previously reported [11, 12]. A linear combination of retained taxa weighted by respective coefficients was performed to compute the taxonomic score (KO score) of individual patients. Meanwhile, the area under the curve (AUC) was estimated to validate the predictive model.

### 2.4. Statistical Analysis

Data were presented as mean ± standard deviation (SD) for normally distributed data and median (first quartile and third quartile) for non-normally distributed data. The *t*-test or Mann–Whitney test was used to compare two groups with normally or non-normally distributed data. Qualitative data were carried out using the *χ*^2^ test for between-group comparisons. Statistical analyses were performed with SPSS version 25.0 (IBM Corp., Armonk, New York). Differential abundance of genera and Kyoto Encyclopedia of Genes and Genomes (KEGG) orthology was tested based on the Wilcoxon rank-sum test, and *P* values were corrected for multiple testing with the Benjamini and Hochberg method. Statistical analyses were conducted using the R software (version 2.15.3). Partial least-squares discriminant analysis (PLS-DA) was carried out using the SIMCA-P software to cluster sample plots across groups. Mediation analysis was used to examine the proposed indirect effects via bootstrapping conducted in the SmartPLS 3 software [13, 14]. Pearson's and Spearman's correlation coefficients were calculated in the R software (version 2.15.3). All statistical analyses were two-sided, and *P* < 0.05 was regarded as statistically significant.

## 3. Results

### 3.1. Baseline Clinical Characteristics of the Participants

Fifty nonvalvular AF patients (32 men, 64%), including 18 low-CHA2DS2-VASc score patients and 32 high-CHA2DS2-VASc score patients, were included in the study. Comparisons of the baseline data are presented in Table 1. Compared to the low CHA2DS2-VASc score group, AF patients in the high CHA2DS2-VASc score group possessed more elderly, HTN, and vascular disease, and lower serum total cholesterol (TC) levels (all *P* < 0.05). Other baseline clinical factors, including the proportion of paroxysmal AF and the time of AF history, were similar between the two groups (Table 1).

### 3.2. Increased GM Diversity in AF Patients with High-CHA2DS2-VASc Scores

**Figure 1:**
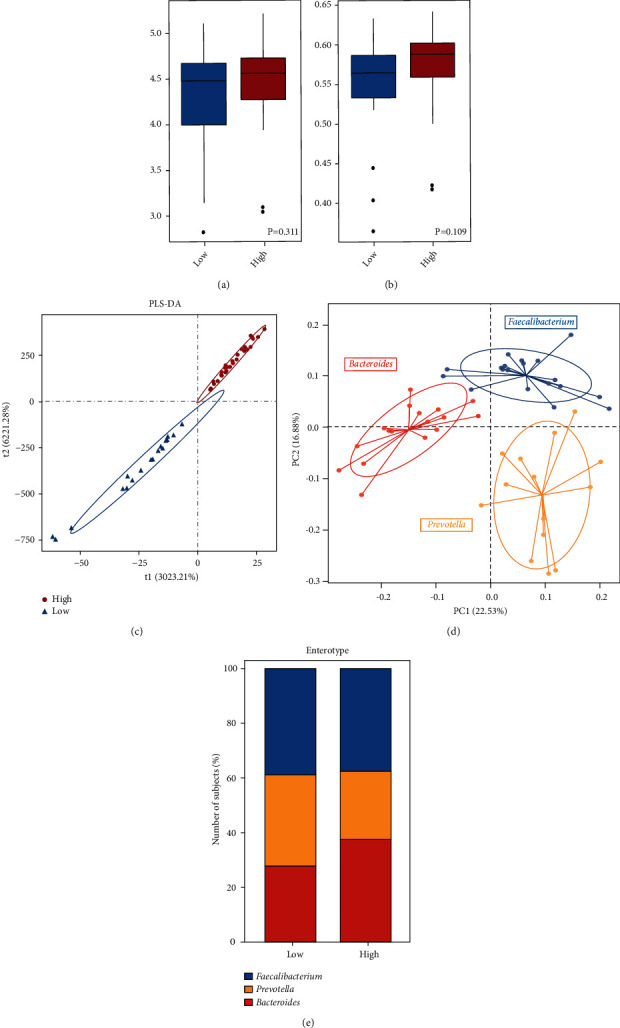
GM in the high or low CHA2DS2-VASc score group. (a, b) Comparison of the microbial *α* diversity comprising Shannon index and Pielou evenness according to the genera profiles in two groups. The boxes represent the interquartile ranges, and the line inside represents the median. (c) A discrepancy in GM composition accessed by PLS-DA. (d) All samples classified into three enterotypes showing significantly different genera (*Bacteroidetes*, *Prevotella,* and *Faecalibacterium*) in the three enterotypes. *P*=2.23*e* − 07, *P*=3.91*e* − 04, and *P*=2.25*e* − 05, respectively. (e) The distribution of enterotypes was different between the two groups. Low *vs*. high; 38.89% *vs.* 37.50% for the enterotype characterized by *Faecalibacterium*; 33.33% *vs.* 25.00% for the enterotype characterized by *Prevotella*; 27.78% *vs.* 37.50% for the enterotype characterized by *Bacteroides*; *P*=0.736, *χ*^2^ test.

It has been demonstrated that microbial diversity is associated with different diseases [15]. Shannon index and Pielou evenness based on the genera profile were calculated to estimate the within-sample (*α*) diversity (*P*=0.311 for Shannon index, Figure 1(a); *P*=0.109 for Pielou's evenness, Figure 1(b)). Due to the small sample size, there was no statistical difference between the two groups. However, the *α* diversity at the genus level was higher in AF patients with high-CHA2DS2-VASc scores. As shown in Figure 1(c), PLS-DA showed a notable discrepancy in gut bacterial composition between the two groups.

Furthermore, the PCoA analysis based on the Jensen–Shannon divergence suggested a significantly altered distribution of enterotypes in the high-CHA2DS2-VASc score group. In comparison, the high-CHA2DS2-VASc score group had an increasing tendency of enterotype 1 dominated by *Bacteroides* and a decreasing tendency of enterotype 2 and 3 dominated by *Prevotella* and *Faecalibacterium,* respectively (Figures 1(d) and 1(e)).

### 3.3. Compositional Alteration of GM in AF Patients with Low or High CHA2DS2-VASc Scores

**Figure 2:**
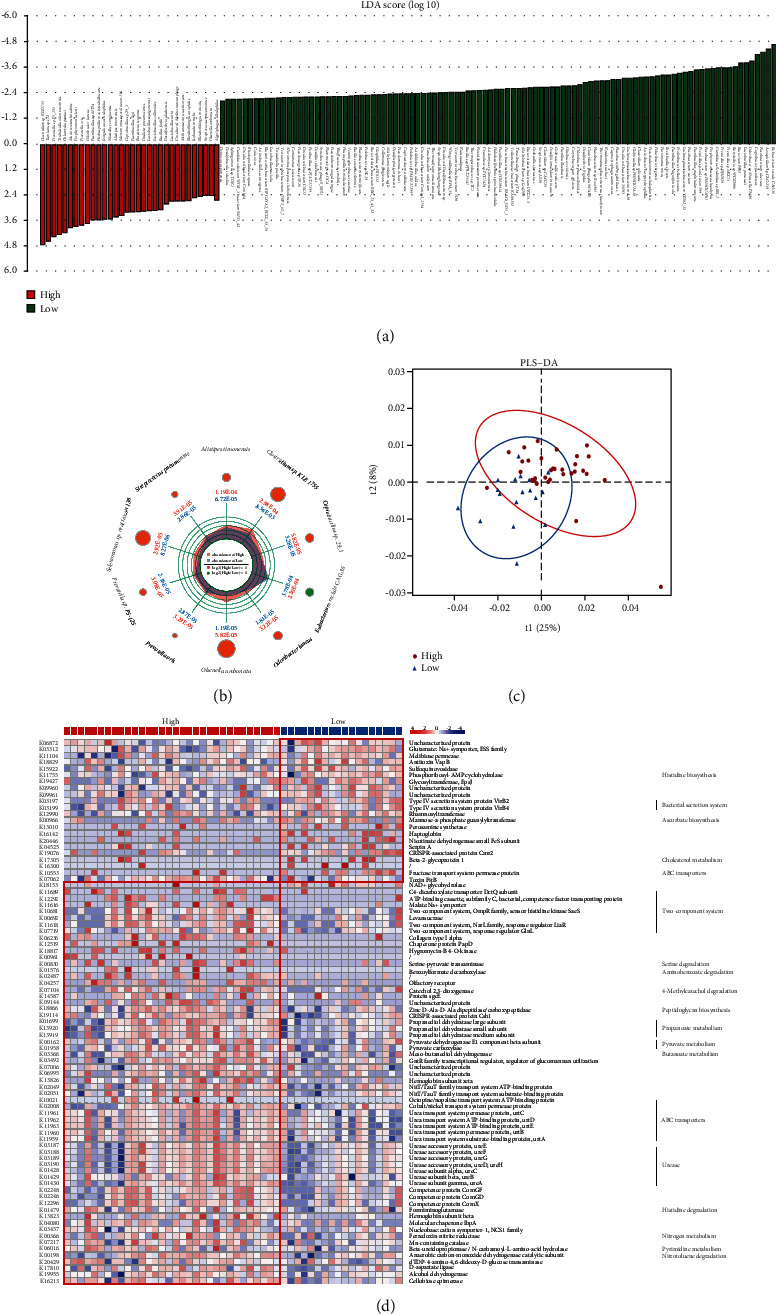
Alterations of GM and related function in the high or low CHA2DS2-VASc score group. (a) LEfSe analysis revealed significant GM differences between the low (green) and high (red) CHA2DS2-VASc score groups at the threshold of absolute LDA score (log 10) >2.0. (b) Radar map presented the relative abundance and multiple in two groups of the top 10 species differentially enriched in AF patients with high CHA2DS2-VASc scores. Wilcoxon rank-sum test, adjust *P* < 0.05. (c, d) Annotation of GM function according to KEGG database between two groups. (c) PLS-DA showing a difference in the two groups. (d) The heat map revealed the shift in the relative abundance (log 10) of KOs across the two groups, and the potential functions of the KOs were presented on the right.

To get an overview of the species composition in two groups, we analyzed the relative abundance of 5436 reference genomes previously annotated [2]. Based on Wilcoxon rank-sum (adjust *P* value <0.05) [10, 16], we found that 132 species were differently enriched between the two groups. 32 species were enriched in the high-CHA2DS2-VASc score group and 100 in low CHA2DS2-VASc score group based on linear discriminant analysis effect size (LEfSe) analysis, all of which had an absolute LDA score (log 10) >2 (Figure 2(a)). The top 10 species differentially dominated in AF patients with high CHA2DS2-VASc scores included *Eubacterium rectale CAG:36, Clostridium* sp. *KLE 1755, Alistipes timonensis, Coprobacillus* sp. *29_1, Streptococcus pneumoniae, Odoribacter laneus, Olsenella umbonata, Prevotella* sp. *P5-125, Prevotella oris*, and *Selenomonas sp. oral taxon 136* (Figure 2(b)). *Eubacterium rectale CAG:36* was significantly reduced in the high-CHA2DS2-VASc score group compared to the low-CHA2DS2-VASc score group (all *P* < 0.05). *Eubacterium rectale (E.rectale)* has critical protective roles against inflammation by producing butanoate, a known anti-inflammatory compound; its abundance is negatively correlated with inflammation levels [17, 18]. Similarly, our observations showed that the higher thromboembolic risk was linked to a reduction in its abundance. These results suggested the potential role of GM dysbiosis in AF patients with high thromboembolic risk.

### 3.4. Functional Variation in GM of High CHA2DS2-VASc Score Patients

According to our data, the gut bacterial composition exhibited obvious distinction in AF patients with high or low CHA2DS2-VASc scores. To figure out the specific biological effect of the GM, we performed corresponding functional annotation based on the KEGG database. According to Wilcoxon rank-sum test and Benjamin and Hochberg test, 84 KEGG orthologys (KOs) were differently enriched between two groups, and PLS-DA revealed the significant discrepancy (Figure 2(c)). Our results showed that in the KEGG pathways, differentially enriched gut bacterial functions related to urease, short-chain fatty acids (SCFAs) (including propanoate and butanoate) metabolism, pyruvate metabolism, amino acid and aromatic compounds metabolism, biosynthesis of ascorbate, degradation of nitrotoluene and aminobenzoate, ABC transporters, two-component system, and so on, were related to the host health (Figure 2(d)). Notably, the sixty-two KOs involved in the metabolism of SCFAs, degradation of histidine, serine, 4-methylcatechol, nitrotoluene, and aminobenzoate, and activation of bacterial urease were overexpressed in patients with high CHA2DS2-VASc scores (all *P* < 0.05). In contrast, the twenty-two KOs involved in the biosynthesis of ascorbate and histidine were distinctly enriched in AF patients with low CHA2DS2-VASc scores (all *P* < 0.05). This gut microbial dysfunction is generally related to many diseases, especially cardiovascular disease [15, 19]. Although functional annotation analysis was predictive, our results preliminarily suggested that altered GM functions might disturb host physiological functions and lead to a high thromboembolic risk in AF patients.

### 3.5. Prediction of High Thromboembolic Risk Based on GM

**Figure 3:**
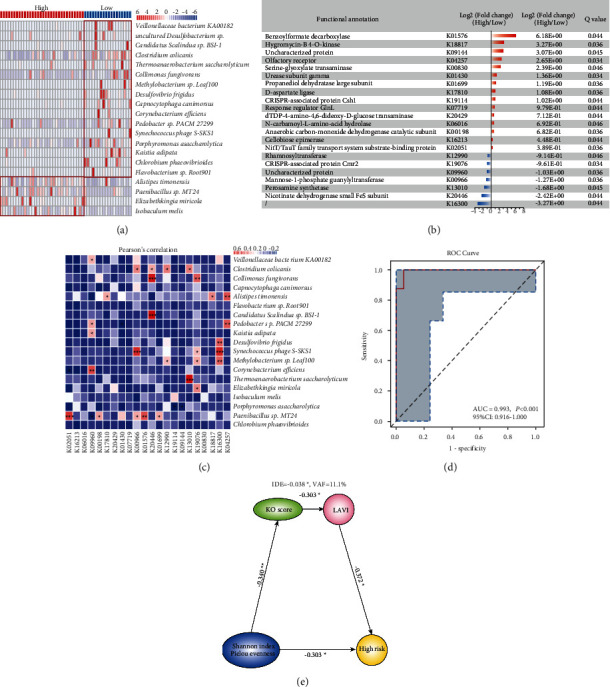
Prediction score of thromboembolic risk in AF patients. (a, b) The most predictive tax of GM and KOs were presented, including 20 species and 22 KOs. (a) The heatmap shows the relative abundance (log 10) of 20 species (all *P* < 0.05). (b) The fold change (log 2) in the relative abundance of 22 KOs. (c) Correlations between 20 species and 22 KOs. Pearson's correlation coefficients were calculated. Red, positive correlation; blue, negative correlation. (d) ROC curve showed the relevant predictive value of the KO score, AUC = 0.993, 95% CI: 0.916–1.000, *P* < 0.001. (e) Mediation analysis on microbial diversity (Shannon index and Pielou's evenness), KO scores, LAVI, and thromboembolic risk of AF patients. VAF = 11.1%, *P* < 0.05. ^*∗*^*P* < 0.05;  ^*∗∗*^*P* < 0.01;  ^*∗∗∗*^*P* < 0.001.

LASSO analysis was used to determine the most predictive tax of GM and KOs, including 20 species and 22 KOs (Figures 3(a) and 3(b)). Pearson's correlation analysis was carried out to evaluate the associations between these species and KO. Notably, predictively KOs were highly correlated with several gut species (Figure 3(c)). Considering the effect of GM relying on its function and aberrant function profiles between two groups, we sought to construct a related grading approach, based on KOs with significant difference enrichment in two groups, to further estimate individualized thromboembolic risk. The KO score was determined by a linear combination of retained taxa weighted by the corresponding coefficients, respectively (Additional file 1: Table S1). AF patients in two groups revealed a significant difference in the KO scores (*P* < 0.001) (Figure S1A), and the KO score had a significant association with thromboembolic risk (*r* = −0.820, *P* < 0.001). The multiple linear regression analysis showed that the KO score as the dependent variable was significantly affected by thromboembolic risk in AF, independently of age, HTN, vascular disease, and TC (adjusted *R*^2^ = 0.679, beta coefficient = −0.759, *P* < 0.001) (Additional file1: Table S2). Then, to assess the predictive value of the KO score, the AUC based on the receiver operating characteristic (ROC) curve was determined (AUC = 0.993, 95%CI: 0.916–1.000, *P* < 0.001) (Figure 3(d)). Our data revealed that the altered GM was significantly associated with thromboembolic risk in AF.

### 3.6. Association between Altered GM and Left Atrial Enlargement

Previous studies have reported an independent association between left atrial volume index (LAVI) and cardioembolic stroke [20, 21]. Similarly, LAVI was positively related to thromboembolic risk in AF in the current study (ROC analysis, AUC = 0.716, 95% CI: 0.565–0.838, *P*=0.023; Spearman's correlation analysis, *R* = 0.354, *P*=0.015) (Figure S1B). Pearson's correlation analysis confirmed the correlation between the KO score and LAVI (*R* = −0.303, *P*=0.038) (Figure S1C). However, *P* wave duration (PWD), as an electrocardiographic (ECG) index reflecting atrial conduction [22], was not significantly associated with the KO score (*R* = −0.172, *P*=0.411) in patients with paroxysmal AF. Furthermore, we performed mediation analysis between the microbial diversity based on Shannon index and Pielou evenness, KO scores, LAVI, and risk of embolization in AF. The results suggested that gut bacterial dysbiosis had an important role in accelerating thromboembolic risk in AF patients, and simultaneously, aberrant microbial function and LAVI together mediated partial indirect effect (VAF = 11.1%, *P* < 0.05) (Figure 3(e)).

## 4. Discussion

GM has a crucial role in multiple physiological functions and metabolism. Alterations in GM profiles are closely associated with various host diseases such as HTN, coronary artery disease, and DM [4, 19]. Our previous study demonstrated that intestinal microbiota dysbiosis contributes to AF development and a higher risk of recurrence following radiofrequency ablation [2, 10].

In this study, we revealed that an increasing degree of disordered GM was associated with higher thromboembolic risk. Meanwhile, significant imbalanced GM functions were also observed, suggesting a possible role of GM dysbiosis and relevant functional variation in increasing thromboembolic risk in AF patients. The newly defined score based on KOs in the current work was significant related to thromboembolic risk in AF. Moreover, mediation analysis revealed that in addition to direct effects, GM dysbiosis via a synergistic effect between the variation in KO and LAVI indirectly increased thromboembolic risk (around 11.1% of this effect). An independent association between left atrial volume index (LAVI), an indicator of the degree of left atrial fibrosis, and cardioembolic stroke in AF has been demonstrated [20, 21, 23]. Hence, gut microbial profile and function were associated with the thromboembolic risk stratification in AF.

Previous studies suggested that *Prevotella* enterotype triggers beneficial effects in lipid metabolism and cardiometabolic diseases, while *Bacteroides* enterotype is associated with systemic inflammation [24, 25]. In the analysis of sequencing data, we found higher levels of *Bacteroides* and lower levels of *Prevotella* in the high-risk CHA2DS2-VASc score group compared to the low-risk CHA2DS2-VASc score group, which was consistent with previous studies examining persistent AF and end-stage renal disease (ESRD) patients [9, 26]. Simultaneously, *Eubacterium rectale* with an anti-inflammatory activity was significantly abundant in the low CHA2DS2-VASc score group. This conversion suggests the association between thromboembolic risk in AF patients and GM.

Short-chain fatty acids produced by intestinal bacterial fermentation of dietary fibers, including acetate, butanoate, and propionate, exert crucial protective effects in cardiac hypertrophy, fibrosis, vascular dysfunction, atherosclerotic, cardiac ventricular arrhythmias, and inflammation [27, 28]. *Prevotella, Lachnospiraceae,* and *Eubacterium rectale* have been associated with the production of SCFAs [7, 29, 30]. In this study, these GM were significantly enriched in the low CHA2DS2-VASc score group. Meanwhile, through functional annotation of the metagenome, we also found that the metabolic functions of SCFAs are altered in AF patients with low or high thromboembolic risk. Our results tentatively indicated that alteration of GM might raise thromboembolic risk via alteration of SCFAs metabolism, but further confirmation are needed. Additionally, recent studies suggested a strong link between microbial production of free amino acids and diseases, and that GM participate in de novo synthesis of several nutritionally essential amino acids, which regulate amino acid homeostasis in the host [31–33]. Wang et al. found that the concentration of serine produced by gut bacteria was decreased in Alzheimer's disease patients [32].

Histidine is a dietary essential amino acid that regulates reactive oxygen scavenging, proton buffering, erythropoiesis, and anti-inflammation [34]. In agreement with our observations, factors related to the degradation of serine and histidine were enriched in AF patients with high thromboembolic risk. Conversely, enzymes engaged in the biosynthesis of histidine were abundant in low thromboembolic risk patients.

Previous research studies reported that urease as a virulence factor of various pathogenic bacteria is related to the progress of several long-lasting diseases, including colitis, atherosclerosis, and rheumatoid arthritis [33, 35]. Similarly, assimilatory ferredoxin-dependent nitrate reductase (nirA) is essential for the full virulence of various bacteria [36, 37]. In the light of our results, three distinct subunits with alpha, beta, and gamma (as a crucial structural component of urease [35, 36]) and nirA were enriched in AF patients with high thromboembolic risk. Several studies demonstrated that 4-methylcatechol, a flavonoid metabolite formed by GM, with potent vasorelaxant, anti-inflammatory, antidiabetic, and antiplatelet effects, reduces endothelial dysfunction [38, 39]. Our data indicated that catechol 2,3-dioxygenase (catE), which was significantly involved in the degradation of 4-methylcatechol, was enriched in AF patients with a high CHA2DS2-VASc score. Ascorbate possesses various protective cardiovascular effects, ranging from anti-oxidative and anti-inflammatory, and may decrease plasma levels of tissue plasminogen activator (tPA) and von Willebrand factor (vWF) as well as affect thrombosis/fibrinolysis system in patients with type 2 diabetes and coronary artery disease [40, 41]. In our study, AF patients with low thromboembolic risk had a higher level of mannose-1-phosphate guanylyltransferase (GMPP) that participate in the biosynthesis of ascorbate. These findings preliminarily indicated the association between gut bacterial dysfunctions and thromboembolic risk of AF patients. Although the functional annotation analyses are predictive, they indicated that the impairment of GM might evoke a disease-linked state through the interference of physiological, metabolic functions.

There were some limitations in this study. The CHA2DS2-VASc score was a surrogate risk marker of thromboembolism, and the sample size was small. Moreover, the duration and type of AF were also associated with thromboembolic risk [23, 42], and the effect of AF duration was not evaluated. Therefore, further studies with a large sample size based on the thromboembolic event, and prospective cohort studies are needed.

## 5. Conclusion

To sum up, our study indicated that the distinct GM dysbiosis and related dysfunctions are associated with high thromboembolic risk in AF patients. The newly constructed tax score based on KOs was related to the thromboembolic risk in AF patients. These findings provided novel insights for further investigation of the interaction between GM and thromboembolic risk. Yet, larger follow-up studies are needed to further confirm and investigate the specific mechanisms involved.

## Figures and Tables

**Table 1:** Clinical characteristics of all subjects.

	Low CHA2DS2-VASc score	High CHA2DS2-VASc score	*P* value (low *vs.* high)
Number	18	32	
Score	0.8 ± 0.8	3.7 ± 1.2	
Congestive heart failure	0	0	
Male/female	12/6	20/12	1.000
Age, years	54.0 ± 9.2	69.7 ± 7.1	<0.001
HTN (%)	2 (11.1)	25 (78.1)	<0.001
DM (%)	2 (11.1)	10 (31.3)	0.170
Stroke/TIA prior (%)	0 (0.0)	4 (12.5)	0.283
Vascular disease (%)	3 (16.7)	29 (90.6)	<0.001
pAF/psAF	13/5	17/15	0.237
Time of AF history, years	0.7 (0.2, 2.5)	1.5 (0.2, 5.8)	0.460
BMI, kg/m^2^	26.3 (24.0, 29.3)	26.5 (23.6, 28.4)	0.952
LVEDD, mm	47.3 ± 3.6	47.2 ± 3.7	0.938
LVESD, mm	28.5 (27.5, 31.3)	29.0 (27.0, 33.0)	0.654
LVEF (%)	67.7 ± 5.0	66.6 ± 6.3	0.548
LAE (%)	9 (50.0)	21 (65.6)	0.242
TC, mmol/L	4.6 ± 1.0	3.8 ± 1.0	0.009
LDL-C, mmol/L	2.6 ± 0.7	2.2 ± 0.9	0.073
HDL-C, mmol/L	1.1 ± 0.4	1.1 ± 0.3	0.950
TG, mmol/L	3.1 ± 5.4	1.4 ± 0.5	0.185
AST, U/L	22.0 ± 12.6	20.6 ± 5.8	0.600
ALT, U/L	26.9 ± 15.9	21.5 ± 10.5	0.201
TBil, *μ*mol/L	12.2 (10.1, 20.3)	14.6 (10.0, 26.5)	0.664
sCr, *μ*mol/L	69.7 ± 13.3	72.9 ± 19.5	0.531
UA, *μ*mol/L	341.3 ± 71.5	327.0 ± 68.6	0.489
WBC, ×10^9^/L	5.7 ± 1.6	6.3 ± 1.4	0.206
HGB, g/L	141.9 ± 17.1	135.0 ± 13.5	0.122
PLT, ×10^9^/L	218.2 ± 46.7	213.6 ± 48.7	0.748
ALB, g/L	41.2 ± 2.9	39.5 ± 2.9	0.055
FBG, mmol/L	4.9 (4.4, 5.6)	5.2 (4.6, 6.0)	0.322
HbA1c (%)	6.0 ± 0.9	6.3 ± 0.7	0.348

Data are expressed as mean ± SD, median (first quartile, third quartile). AF, atrial fibrillation; ALT, alanine aminotransferase; AST, aspartate aminotransferase; ALB, albumin; BMI, body mass index; DM, diabetes mellitus; FBG, fasting blood glucose; HTN, hypertension; HGB, hemoglobin; HbA1c, hemoglobin A1c; HDL-C, high-density lipoprotein cholesterol; LVEDD, left ventricular and diastolic diameter; LVESD, left ventricular and systolic systolic diameter; LVEF, left ventricular ejection fraction; LAE, left atrial enlargement, pAF, paroxysmal atrial fibrillation; psAF, persistent atrial fibrillation; PLT, platelet; sCr, serum creatinine; TC, total cholesterol; TG, triglyceride; TIA, transient ischemic attack; LDL-C, low-density lipoprotein cholesterol; TBil, total bilirubin; UA, uric acid, WBC white blood cell.

## Data Availability

The datasets analyzed during the current study are available in the EMBL European Nucleotide Archive (ENA) under the BioProject accession code PRJEB28384.

## Supplementary Materials

Supplementary Materials Additional file 1: supplementary methods of metagenomic analyses. Additional file 1. Table S1: the model and score of the KO score. Additional file 1. Table S2: linear regression analysis of the KO score. Additional file 1. Figure S1: (A) comparison of the KO scores in two groups. Wilcoxon rank-sum test; ^*∗∗∗*^*P* < 0.001. (B) ROC curve for assessing the association between LAVI and thromboembolic risk in AF. (C) Pearson's correlation analysis between the KO score and LAVI.
