# Supplementary material for: Association between Gut Microbiota Dysbiosis and the CHA2DS2-VASc Score in Atrial Fibrillation Patients
Source: Int J Clin Pract. 2022 May 6;2022:7942605. doi: 10.1155/2022/7942605 (PMC9159190; doi:10.1155/2022/7942605)
Supplement: Supplementary Materials — Additional file 1: supplementary methods of metagenomic analyses. Additional file 1. Table S1: the model and score of the KO score. Additional file 1. Table S2: linear regression analysis of the KO score. Additional file 1. Figure S1: (A) comparison of the KO scores in two groups. Wilcoxon rank-sum test; ∗∗∗P < 0.001. (B) ROC curve for assessing the association between LAVI and thromboembolic risk in AF. (C) Pearson's correlation analysis between the KO score and LAVI. [file 7942605.f1.docx]

**Analysis between Gut Microbiota dysbiosis and the CHA2DS2-VASc score in atrial fibrillation patients**

**Supplementary Methods**

**Metagenomic analyses**

Paired-end metagenomic sequencing was performed on the Illumina platform (insert size 300 bp, read length 150 bp). After quality control, the reads aligned with the human genome (alignment with Short Oligonucleotide Analysis Package 2 [SOAP2], version 2.21, parameters: −s 135, −l 30, −v 7, −m 200, −x 400) were removed. The assembly of reads was executed by SOAP de novo (version 2.04, parameters: −d 1 −M 3 −R −u −F), and the clean data were mapped against scaffolds by SOAP2 (version 2.21, parameters: −m 200 −x 400 −s 119). Subsequently, gene prediction from the assembled contigs was performed by MetaGeneMark (prokaryotic GeneMark, hidden Markov model version 2.10). A nonredundant gene catalogue was constructed with Cluster Database at High Identity with Tolerance (CD-HIT, version 4.5.8, parameters: −G 0 −aS 0.9 −g 1 −d 0 −c 0.95). Reads were realigned to the gene catalogue with SOAP2 using parameters to determine the abundance of genes. The gene abundance was calculated by counting the number of reads and normalized by gene length. Then, to assess the taxonomic assignment, genes were aligned to the integrated nr database by DIAMOND (version 0.7.9.58, default parameters except that −k 50 −sensitive −e 0.00001). Significant matches for each gene, defined by E values of ≤10× E value of the top hit, were determined, and the retained matches were used to distinguish taxonomic groups. The taxonomical level of each gene was identified according to the lowest common ancestor-based algorithm implemented with MEGAN (MEtaGenome ANalyzer), and the abundance of a taxonomic group was calculated according to the sum abundance of genes annotated to a feature. To assess the function of gut microbiota, all genes in the catalogue were aligned to the KEGG database (release 73.1, with animal and plant genes removed) by DIAMOND (version 0.7.9.58, default parameters except for –k 50 –sensitive –e 0.00001), and each protein was assigned to the KEGG database using the highest-scoring annotated hits containing ≥1 high-scoring segment pair scoring >60 hits. Through summing the abundance of genes annotated to the same feature, the abundance of the KEGG module was calculated.

**Supplementary tables and table legends**

**Table S1**

The model and score of the KO score.

| Sample | The KO score | Group | Sample | The KO score | Group |
| --- | --- | --- | --- | --- | --- |
| AF1 | -0.223311818 | High | AF47 | -2.759942459 | High |
| AF2 | -0.587826847 | High | AF48 | -2.940552695 | High |
| AF3 | -1.665634375 | High | AF50 | -1.483876176 | High |
| AF4 | -1.888287525 | High | AF8 | 0.530376238 | Low |
| AF5 | -4.093384312 | High | AF9 | 1.16574467 | Low |
| AF6 | -1.557995422 | High | AF11 | 0.942087302 | Low |
| AF7 | -0.965856853 | High | AF12 | 1.634343652 | Low |
| AF10 | -2.036450302 | High | AF13 | 0.969285885 | Low |
| AF14 | -0.465154335 | High | AF20 | 2.54660768 | Low |
| AF15 | -1.452489656 | High | AF21 | 1.161133119 | Low |
| AF16 | -3.880377911 | High | AF22 | 1.478872004 | Low |
| AF17 | -2.698774928 | High | AF23 | -0.601078863 | Low |
| AF18 | -1.82478556 | High | AF24 | 2.616746473 | Low |
| AF19 | -2.9104362 | High | AF28 | 1.262623972 | Low |
| AF25 | -4.031296329 | High | AF34 | 0.242250754 | Low |
| AF26 | -0.964481534 | High | AF39 | 2.090101069 | Low |
| AF27 | -1.260871017 | High | AF40 | 0.369156269 | Low |
| AF29 | -2.433416262 | High | AF41 | 1.075200025 | Low |
| AF30 | -4.979078782 | High | AF42 | 1.103770959 | Low |
| AF31 | -2.615318131 | High | AF45 | 2.177843109 | Low |
| AF32 | -3.119870542 | High | AF49 | 1.089633512 | Low |
| AF33 | -1.516947606 | High |  |  |  |
| AF35 | -4.897781963 | High |  |  |  |
| AF36 | -2.207048889 | High |  |  |  |
| AF37 | -1.544089339 | High |  |  |  |
| AF38 | -2.385001246 | High |  |  |  |
| AF43 | -2.245021757 | High |  |  |  |
| AF44 | -3.285341319 | High |  |  |  |
| AF46 | -0.551544015 | High |  |  |  |

The KO score = [2.3024 × (Intercept)] + [-4205.7017 × K02051] + [-66.6711 × K16213] + [-12836.5277 × K06016] + [15532.5178 × K09960] + [-22040.3454 × K00198] + [-17056.7303 × K17810] + [-36181.4273 × K20429] + [-22890.7181 × K01430] + [-10236.3635 × K07719] + [240575.8302 × K00966] + [-92725.3121 × K01576] + [1226531.3513 × K20446] + [-123079.3580 × K01699] + [1688.9355 × K12990] + [-114461.7498 × K19114] + [-17409.5866 × K09144] + [99882.2351 × K13010] + [752247.7397 × K19076] + [-142869.0097 × K00830] + [-706043.2601 × K18817] + [759419.1678 × K16300] + [-4431144.1631 × K04257].

**Table S2**

Linear regression analysis of the KO score.

|  | Unstandardized  coefficients | Standardized  coefficients | *P* value |
| --- | --- | --- | --- |
|  | B (95% CI) | Beta |  |
| (Constant) | -0.497 (-3.524, 2.531) |  | 0.743 |
| Thromboembolic risk | -3.129 (-4.443, -1.814) | -0.759 | <0.001 |
| Age | 0.018 (-0.026, 0.063) | 0.100 | 0.406 |
| HTN | -0.127 (-0.990, 0.736) | -0.032 | 0.768 |
| Vascular disease | -0.517 (-1.590, 0.555) | -0.125 | 0.336 |
| TC | 0.177 (-0.162, 0.515) | 0.093 | 0.299 |

HTN, hypertension; TC, total cholesterol.

**Supplementary figures and figure legends**

**
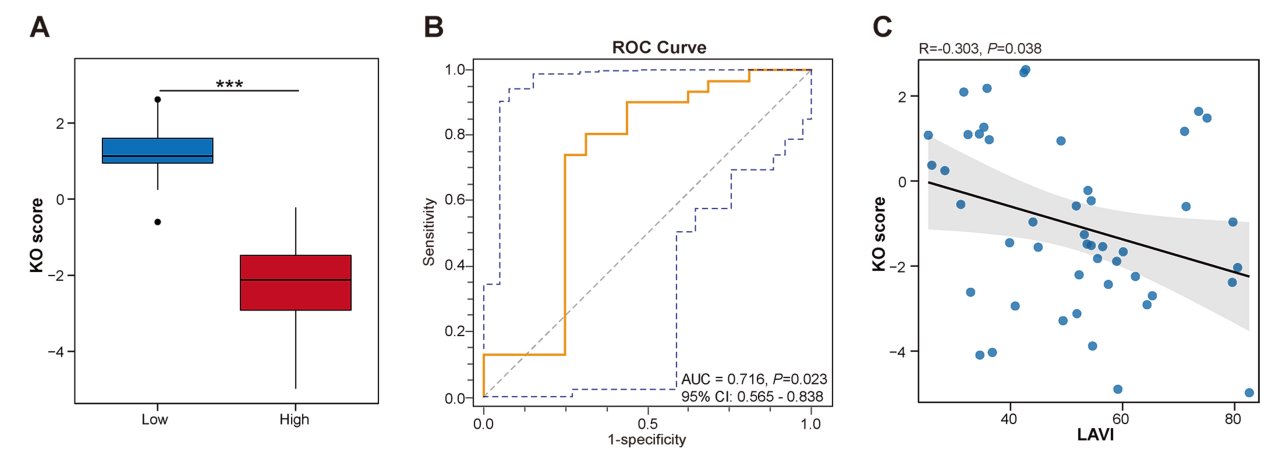
**

**Figure S1. (A)** Comparison of the KO scores in two groups. Wilcoxon rank-sum test; ***, *P*<0.001. **(B)** ROC curve for assessing the association between LAVI and thromboembolic risk in AF. **(C)** Pearson’s correlation analysis between the KO score and LAVI.
